# Supplementary material for: Genomics of Experimental Adaptive Radiation in the Cryptic Coloration of Feather Lice
Source: Genome Biol Evol. 2025 May 7;17(5):evaf083. doi: 10.1093/gbe/evaf083 (PMC12092913; doi:10.1093/gbe/evaf083)
Supplement: evaf083_Supplementary_Data [file evaf083_supplementary_data.zip › supplement_revision1.docx]

## **SUPPLEMENTARY MATERIALS**

**p*F*_ST_ uses a likelihood ratio test to identify sites differentiated between populations**

A full description of p*F*_ST_ is available in Kronenberg et al. (2015). Traditional *F*_ST_ statistics can produce false positive signals of differentiation at loci with low sequencing coverage due to inaccurate measurement of allele frequency. p*F*_ST_ accounts for this problem using a maximum likelihood approach such that only highly differentiated sites with good coverage are identified as significantly divergent between populations. p*F*_ST_ instead identifies differentiated sites through a likelihood ratio contrasting the likelihood that allele frequency of the target population (AFT) is drawn from the same distribution as the the background population (AFB) against the likelihood that AFT is drawn from a different distribution than AFB. The allelic counts in this model come from the counts of sequencing reads representing the two alleles at the locus of interest. Crucially, the two contrasted likelihoods are determined taking into account the likelihood of genotyping errors, thus avoiding the problem faced by traditional *F*_ST_ statistics.

The binomial density function is specified by *n*, *s*, and *p*, respectively, the number of successes, the number of trials, and the underlying probability of a success. Here, *n* corresponds to the number of alternate alleles out of either the number of target population alleles (NT), the number of background alleles (NB), or the combination of both (NC). *K*, similarly, is the total number of alleles in the target (KT), background (KB), or both. Similarly, *p* here is equivalent to AFT, AFB, or AFC. *D* is the likelihood ratio test statistic.


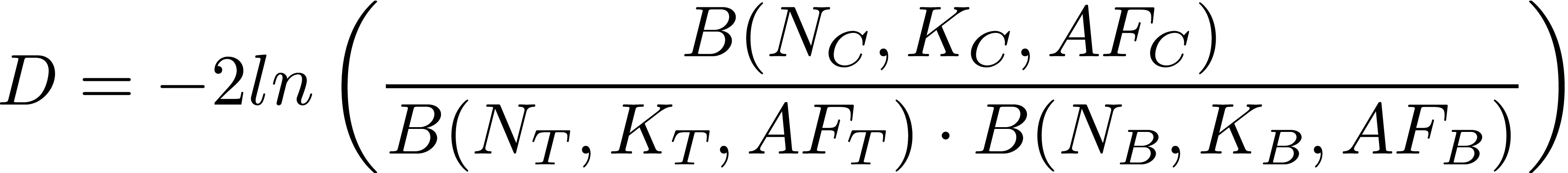


Here, D is a function of N, K, and AF for the target and background alleles. A large D indicates that the null hypothesis should be rejected. Because D is chi-squared distributed, it can be converted to a p-value by chi-squared lookup. The below equations show how to calculate allele counts and frequencies while taking into account genotype likelihood information, where *gl* indicates genotype likelihood, and the subscripts 1, 2, and 3 indicate, respectively, a homozygous A individual, a heterozygote, and a homozygous B individual.


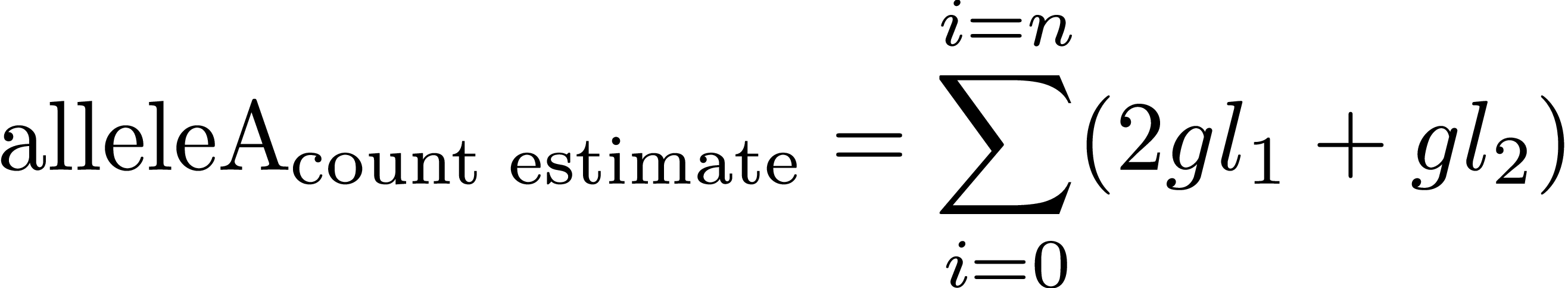


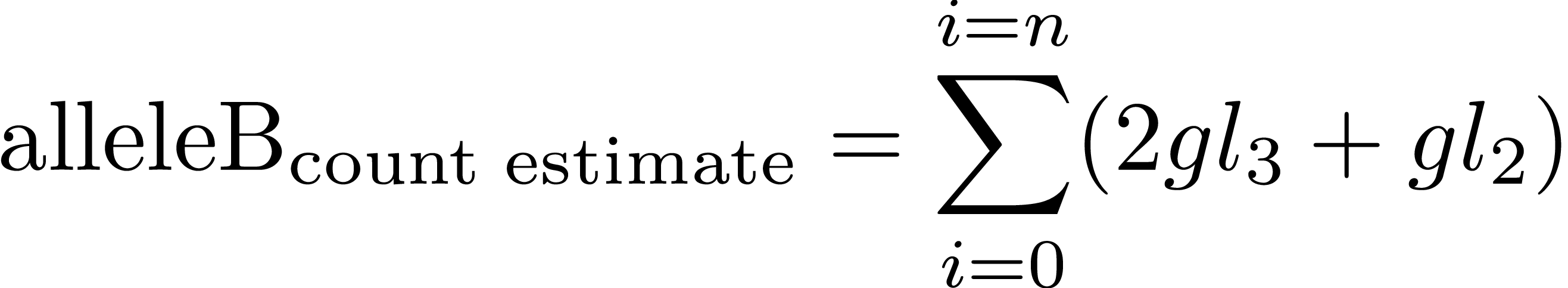


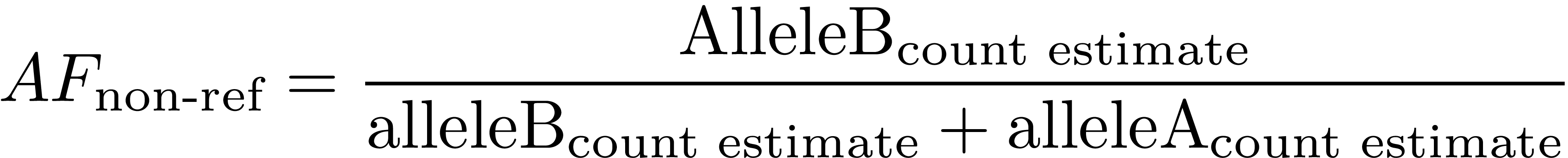


p*F*_ST_ can be run on individually-genotyped or pooled samples. The above equations describe how it is run in the individually-genotyped case. In the case of pooled samples, the only change is that the binomial distributions used in calculating D are replaced with beta distributions with parameters estimated by the method of moments. Here, if the sample mean is x-bar and the sample variance is v-bar, then alpha and beta are calculated as follows:


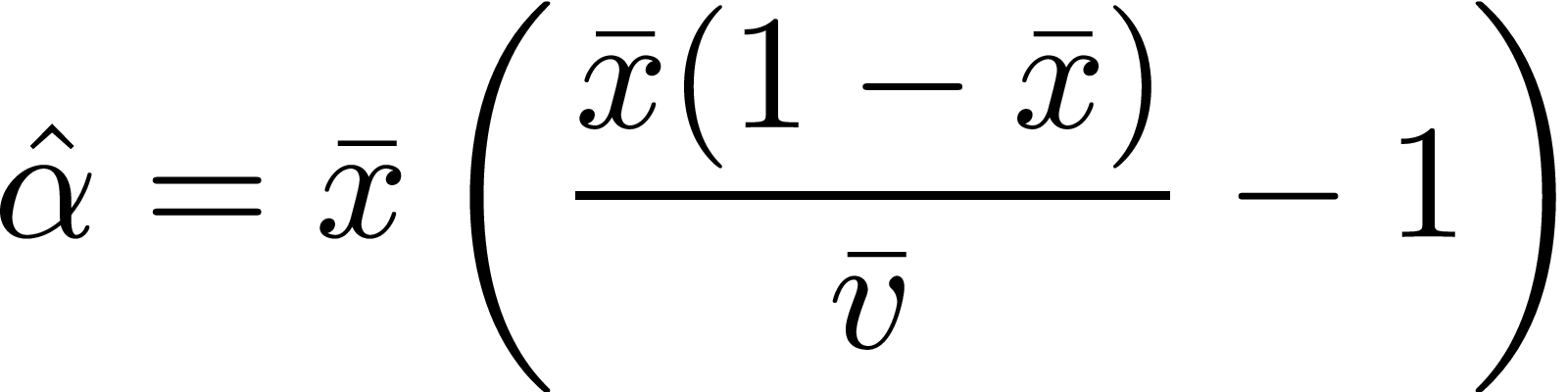


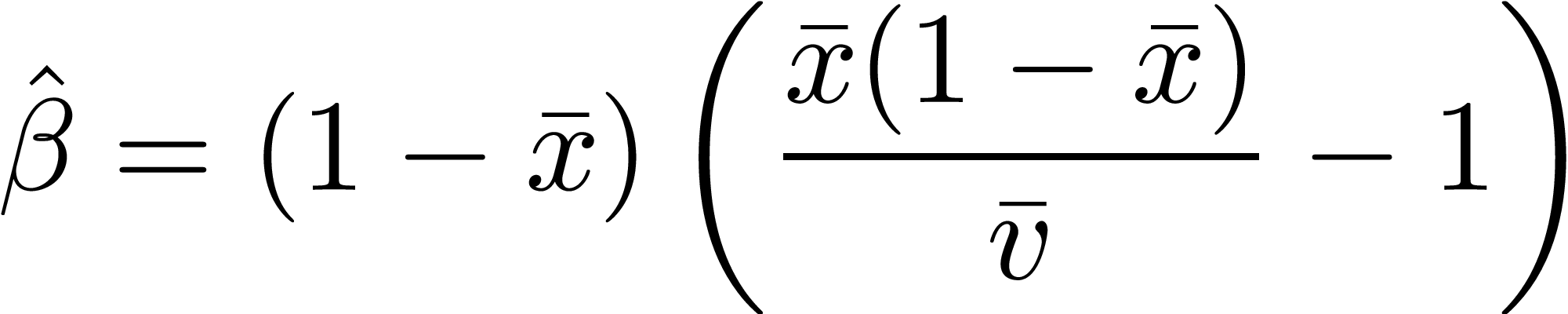


Finally, this means *D* is calculated as follows for the pooled case:


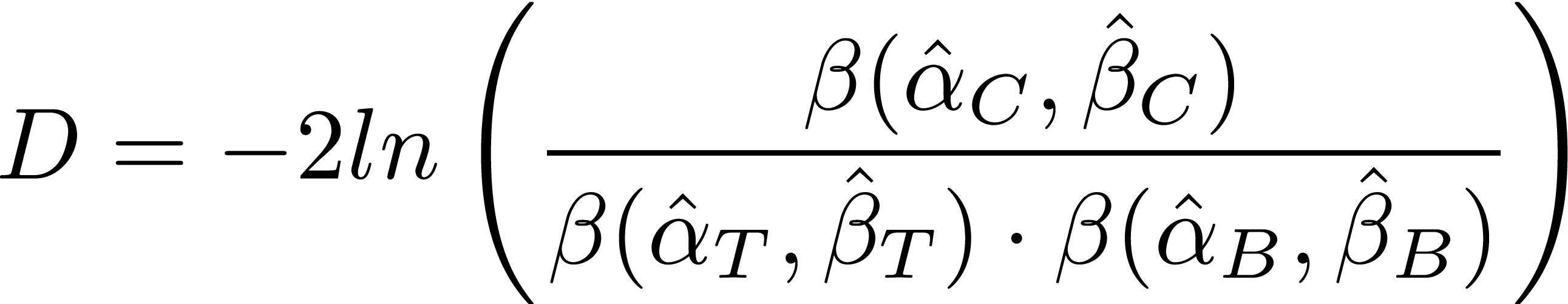


##

## **The evolved populations are largely structured by ancestral relationships**

We used *popvae* (Battey et al. 2021) to identify structure in the evolved populations based on individuals sequenced in the ancestral generation and the 36-month time point. Based on a whole-genome assay of population structure, we found individuals could clearly be grouped by their respective populations (Fig. 3). Because we ran *popvae* on a broad set of polymorphisms drawn from across the genome, we expect differentiation of the populations to be driven primarily by genetic drift and population structure (here, founder effects). While selection should act at a subset of loci, drift acts on the entire genome; thus, measures of population structure should be driven more by drift than by selection, excepting cases where selection is occurring across the entire genome.

Notably, the populations cluster according to their replicate number. This is consistent with the design of the experiment. Each replicate set of treatments was drawn from the initial ancestral population in a manner staggered in time. Replicate 2 was founded six weeks after replicate 1, replicate 3 was founded six weeks after that, and replicate 4 six weeks after that. This was done to make handling the lice more practical. Owing to this, lice from a given replicate should be more closely related to other lice from the same replicate, even if they are from another treatment, than they are to other lice within their own treatment, but from a different replicate.

## SUPPLEMENTARY TABLES

**Supplementary Table 1.** Linkage disequilibrium decays to produce approximately 377 kb haplotypes by 48 generations. The expected distance between recombination is calculated by assuming one recombination event per chromosome per generation, then calculating the distance in basepairs expected by a given number of generations, i.e., (genome size / number of chromosomes) / generations. The recombination rate required is the rate of recombination between two points needed to reduce linkage disequilibrium to 1/3 of its initial value. It is equal to 1 – (final_ld / initial_ld)^(1 / generations). The basepair distance corresponding to recombination rate is simply the recombination rate times the chromosome size, and indicates the average distance between two loci that would have a recombination rate such that linkage disequilibrium would be reduced to 1/3 of its original value.

| Generation | Expected distance between recombination events | Recombination rate required | Basepair distance corresponding to recombination rate |
| --- | --- | --- | --- |
| 6 | 2777778 | 0.1673 | 2788845 |
| 12 | 1388889 | 0.0875 | 1458214 |
| 18 | 925926 | 0.0592 | 986900 |
| 24 | 694444 | 0.0447 | 745793 |
| 30 | 555555 | 0.0360 | 599353 |
| 36 | 462962 | 0.0301 | 500979 |
| 42 | 396825 | 0.0258 | 430343 |
| 48 | 347222 | 0.0226 | 377164 |
| 54 | 308642 | 0.0201 | 335682 |

**Supplementary Table 2.** Permutation tests show that many pairs of replicates have more putative selected sites in common than expected by chance. “Overlap count p” refers to the probability that the selected sites overlapped by at least one basepair by chance. For example, two selected sites that overlap by 100 bp would count as one overlap. “Overlap basepair p” refers to the probability that the selected sites had a number of basepairs overlapping that often or more by chance. For example, two selected sites that overlap by 100 bp would count as 100 bp of overlap. Bolded values have a p-value less than or equal to 0.05.

| Overlap  Count p | Overlap Basepair p | Bird Color | Replicates |
| --- | --- | --- | --- |
| 0.2675 | 0.3390 | White | 3:4 |
| 0.1290 | 0.3530 | White | 1:4 |
| 0.1095 | 0.5300 | White | 2:3 |
| 0.1280 | 0.2570 | White | 1:3 |
| 0.4515 | 0.7500 | White | 1:2 |
| **0.0000** | **0.0000** | White | 2:4 |
| 0.0685 | 0.1160 | Black | 3:4 |
| 0.3930 | 0.3930 | Black | 1:3 |
| **0.0335** | **0.0335** | Black | 2:3 |
| 0.2995 | 0.4995 | Black | 1:4 |
| 0.0735 | 0.0735 | Black | 2:4 |
| **0.0005** | **0.0000** | Black | 1:2 |

**Supplementary Table 3.** The frequency of shared selected sites did not differ significantly between louse populations experiencing selection on white or black pigeons (Fisher’s Exact Test)

| Host treatment: | White Pigeons  Normal Preening | |  | Black Pigeons  Normal Preening | |  |  |  |
| --- | --- | --- | --- | --- | --- | --- | --- | --- |
|  | total loci | pairwise overlaps |  | total loci | pairwise overlaps | p-value | 95% CI Min. | 95% CI Max. |
|  | 71 | 20 |  | 32 | 3 | 0.121 | 0.798 | 16.786 |

**Supplementary Table 4.** Putative selected sites have significantly higher starting allele frequencies than randomly chosen sites. Here, we take 65 polymorphisms putatively under selection in lice on white pigeons and 26 polymorphisms putatively under selection in lice on black pigeons and compare the starting allele frequencies of these sites with the same sites in lice on preening-impaired birds. We also compared the starting allele frequencies of putatively selected sites to randomly chosen sites in lice on normally preening birds. The starting minor allele frequency of putatively selected sites from lice on normally preening birds is not significantly different than the same sites from lice on impaired-preening birds; however, these sites do differ significantly in starting minor allele frequency from randomly chosen sites. The results of these comparisons are consistent with the expectation that polymorphisms with high minor allele frequencies are more sensitive to selection (Gravel 2016). We used Tukey’s HSD test to determine which pairwise comparisons were significant. Bolded values have a p-value less than or equal to 0.05.

| Treatment | Comparison | | | p Adjusted |
| --- | --- | --- | --- | --- |
|  | Normal preening | Impaired preening | Randomly chosen sites, normal preening |  |
| White pigeon | X | X |  | 1 |
| White pigeon | X |  | X | **0** |
| Black pigeon | X | X |  | 1 |
| Black pigeon | X |  | X | **0.038** |

**Supplementary Table 5.** Gene ontology categories enriched in selected loci. FDR BH here is false discovery rate correction by the Benjamini-Hochberg method. BP is biological process, MF is molecular function, and CC is cellular component

| Host Color | GO | GO hierarchy | Function | Ratio in study | Ratio in annotation | p Uncorrected | p FDR BH |
| --- | --- | --- | --- | --- | --- | --- | --- |
| White | GO:0007606 | BP | sensory perception of chemical stimulus | 15/2400 | 26/13362 | 6.53E-06 | 0.0115 |
| White | GO:0050877 | BP | nervous system process | 15/2400 | 30/13362 | 6.35E-05 | 0.0240 |
| White | GO:0007600 | BP | sensory perception | 15/2400 | 30/13362 | 6.35E-05 | 0.0240 |
| White | GO:0003008 | BP | system process | 15/2400 | 30/13362 | 6.35E-05 | 0.0240 |
| White | GO:0007608 | BP | sensory perception of smell | 9/2400 | 13/13362 | 6.82E-05 | 0.0240 |
| White | GO:0032501 | BP | multicellular organismal process | 24/2400 | 62/13362 | 0.000161 | 0.0471 |
| White | GO:0004984 | MF | olfactory receptor activity | 9/2400 | 13/13362 | 6.82E-05 | 0.0207 |
| White | GO:0004252 | MF | serine-type endopeptidase activity | 3/2400 | 110/13362 | 1.25E-06 | 0.00152 |
| White | GO:0017171 | MF | serine hydrolase activity | 6/2400 | 126/13362 | 1.89E-05 | 0.00766 |
| White | GO:0008236 | MF | serine-type peptidase activity | 6/2400 | 126/13362 | 1.89E-05 | 0.00766 |
| Black | GO:0005890 | CC | sodium:potassium-exchanging ATPase complex | 5/1109 | 8/13362 | 0.000177 | 0.0352 |
| Black | GO:0090533 | CC | cation-transporting ATPase complex | 5/1109 | 8/13362 | 0.000177 | 0.0352 |

**Supplementary Table 6.** Counts of sequenced individuals from each population and time point.

| Preening | Host | Replicate | Time point (months) | Count |
| --- | --- | --- | --- | --- |
| Preening Impaired | Black Homer | 1 | 6 | 50 |
| Preening Impaired | Black Homer | 1 | 12 | 50 |
| Preening Impaired | Black Homer | 1 | 18 | 50 |
| Preening Impaired | Black Homer | 1 | 24 | 26 |
| Preening Impaired | Black Homer | 1 | 30 | 50 |
| Preening Impaired | Black Homer | 1 | 36 | 50 |
| Preening Impaired | Black Homer | 1 | 42 | 50 |
| Preening Impaired | Black Homer | 1 | 48 | 50 |
| Preening Impaired | Black Homer | 2 | 6 | 50 |
| Preening Impaired | Black Homer | 2 | 12 | 50 |
| Preening Impaired | Black Homer | 2 | 18 | 50 |
| Preening Impaired | Black Homer | 2 | 24 | 26 |
| Preening Impaired | Black Homer | 2 | 30 | 50 |
| Preening Impaired | Black Homer | 2 | 36 | 50 |
| Preening Impaired | Black Homer | 2 | 42 | 50 |
| Preening Impaired | Black Homer | 2 | 48 | 50 |
| Preening Impaired | Black Homer | 3 | 6 | 50 |
| Preening Impaired | Black Homer | 3 | 12 | 50 |
| Preening Impaired | Black Homer | 3 | 18 | 50 |
| Preening Impaired | Black Homer | 3 | 24 | 24 |
| Preening Impaired | Black Homer | 3 | 30 | 49 |
| Preening Impaired | Black Homer | 3 | 36 | 50 |
| Preening Impaired | Black Homer | 3 | 42 | 50 |
| Preening Impaired | Black Homer | 3 | 48 | 48 |
| Preening Impaired | Black Homer | 4 | 6 | 50 |
| Preening Impaired | Black Homer | 4 | 12 | 50 |
| Preening Impaired | Black Homer | 4 | 18 | 50 |
| Preening Impaired | Black Homer | 4 | 24 | 26 |
| Preening Impaired | Black Homer | 4 | 30 | 51 |
| Preening Impaired | Black Homer | 4 | 36 | 50 |
| Preening Impaired | Black Homer | 4 | 42 | 50 |
| Preening Impaired | Black Homer | 4 | 48 | 50 |
| Preening Impaired | Feral (grey) | 1 | 6 | 50 |
| Preening Impaired | Feral (grey) | 1 | 12 | 50 |
| Preening Impaired | Feral (grey) | 1 | 18 | 49 |
| Preening Impaired | Feral (grey) | 1 | 24 | 25 |
| Preening Impaired | Feral (grey) | 1 | 30 | 50 |
| Preening Impaired | Feral (grey) | 1 | 36 | 50 |
| Preening Impaired | Feral (grey) | 1 | 42 | 50 |
| Preening Impaired | Feral (grey) | 1 | 48 | 50 |
| Preening Impaired | Feral (grey) | 2 | 6 | 50 |
| Preening Impaired | Feral (grey) | 2 | 12 | 50 |
| Preening Impaired | Feral (grey) | 2 | 18 | 50 |
| Preening Impaired | Feral (grey) | 2 | 24 | 27 |
| Preening Impaired | Feral (grey) | 2 | 30 | 39 |
| Preening Impaired | Feral (grey) | 2 | 36 | 50 |
| Preening Impaired | Feral (grey) | 2 | 42 | 50 |
| Preening Impaired | Feral (grey) | 2 | 48 | 50 |
| Preening Impaired | Feral (grey) | 3 | 6 | 50 |
| Preening Impaired | Feral (grey) | 3 | 12 | 50 |
| Preening Impaired | Feral (grey) | 3 | 18 | 50 |
| Preening Impaired | Feral (grey) | 3 | 24 | 26 |
| Preening Impaired | Feral (grey) | 3 | 30 | 50 |
| Preening Impaired | Feral (grey) | 3 | 36 | 50 |
| Preening Impaired | Feral (grey) | 3 | 42 | 50 |
| Preening Impaired | Feral (grey) | 3 | 48 | 50 |
| Preening Impaired | Feral (grey) | 4 | 6 | 50 |
| Preening Impaired | Feral (grey) | 4 | 12 | 50 |
| Preening Impaired | Feral (grey) | 4 | 18 | 51 |
| Preening Impaired | Feral (grey) | 4 | 24 | 26 |
| Preening Impaired | Feral (grey) | 4 | 30 | 50 |
| Preening Impaired | Feral (grey) | 4 | 36 | 51 |
| Preening Impaired | Feral (grey) | 4 | 42 | 50 |
| Preening Impaired | Feral (grey) | 4 | 48 | 50 |
| Preening Impaired | White Homer | 1 | 6 | 50 |
| Preening Impaired | White Homer | 1 | 12 | 50 |
| Preening Impaired | White Homer | 1 | 18 | 50 |
| Preening Impaired | White Homer | 1 | 24 | 26 |
| Preening Impaired | White Homer | 1 | 30 | 50 |
| Preening Impaired | White Homer | 1 | 36 | 51 |
| Preening Impaired | White Homer | 1 | 42 | 50 |
| Preening Impaired | White Homer | 1 | 48 | 50 |
| Preening Impaired | White Homer | 2 | 6 | 50 |
| Preening Impaired | White Homer | 2 | 12 | 50 |
| Preening Impaired | White Homer | 2 | 18 | 50 |
| Preening Impaired | White Homer | 2 | 24 | 26 |
| Preening Impaired | White Homer | 2 | 30 | 50 |
| Preening Impaired | White Homer | 2 | 36 | 49 |
| Preening Impaired | White Homer | 2 | 42 | 50 |
| Preening Impaired | White Homer | 2 | 48 | 51 |
| Preening Impaired | White Homer | 3 | 6 | 50 |
| Preening Impaired | White Homer | 3 | 12 | 50 |
| Preening Impaired | White Homer | 3 | 18 | 49 |
| Preening Impaired | White Homer | 3 | 24 | 26 |
| Preening Impaired | White Homer | 3 | 30 | 46 |
| Preening Impaired | White Homer | 3 | 36 | 50 |
| Preening Impaired | White Homer | 3 | 42 | 50 |
| Preening Impaired | White Homer | 3 | 48 | 49 |
| Preening Impaired | White Homer | 4 | 6 | 50 |
| Preening Impaired | White Homer | 4 | 12 | 50 |
| Preening Impaired | White Homer | 4 | 18 | 47 |
| Preening Impaired | White Homer | 4 | 24 | 26 |
| Preening Impaired | White Homer | 4 | 30 | 50 |
| Preening Impaired | White Homer | 4 | 36 | 50 |
| Preening Impaired | White Homer | 4 | 42 | 49 |
| Preening Impaired | White Homer | 4 | 48 | 12 |
| Preening | Black Homer | 1 | 6 | 34 |
| Preening | Black Homer | 1 | 12 | 50 |
| Preening | Black Homer | 1 | 18 | 50 |
| Preening | Black Homer | 1 | 24 | 14 |
| Preening | Black Homer | 1 | 30 | 47 |
| Preening | Black Homer | 1 | 36 | 49 |
| Preening | Black Homer | 1 | 42 | 37 |
| Preening | Black Homer | 1 | 48 | 50 |
| Preening | Black Homer | 2 | 6 | 50 |
| Preening | Black Homer | 2 | 12 | 50 |
| Preening | Black Homer | 2 | 18 | 11 |
| Preening | Black Homer | 2 | 24 | 18 |
| Preening | Black Homer | 2 | 30 | 38 |
| Preening | Black Homer | 2 | 36 | 17 |
| Preening | Black Homer | 2 | 42 | 47 |
| Preening | Black Homer | 2 | 48 | 26 |
| Preening | Black Homer | 3 | 6 | 50 |
| Preening | Black Homer | 3 | 12 | 48 |
| Preening | Black Homer | 3 | 18 | 32 |
| Preening | Black Homer | 3 | 24 | 26 |
| Preening | Black Homer | 3 | 30 | 24 |
| Preening | Black Homer | 3 | 36 | 31 |
| Preening | Black Homer | 3 | 42 | 0 |
| Preening | Black Homer | 3 | 48 | 0 |
| Preening | Black Homer | 4 | 6 | 32 |
| Preening | Black Homer | 4 | 12 | 15 |
| Preening | Black Homer | 4 | 18 | 30 |
| Preening | Black Homer | 4 | 24 | 14 |
| Preening | Black Homer | 4 | 30 | 39 |
| Preening | Black Homer | 4 | 36 | 24 |
| Preening | Black Homer | 4 | 42 | 8 |
| Preening | Black Homer | 4 | 48 | 6 |
| Preening | Feral (grey) | 1 | 6 | 20 |
| Preening | Feral (grey) | 1 | 12 | 16 |
| Preening | Feral (grey) | 1 | 18 | 40 |
| Preening | Feral (grey) | 1 | 24 | 5 |
| Preening | Feral (grey) | 1 | 30 | 34 |
| Preening | Feral (grey) | 1 | 36 | 20 |
| Preening | Feral (grey) | 1 | 42 | 17 |
| Preening | Feral (grey) | 1 | 48 | 3 |
| Preening | Feral (grey) | 2 | 6 | 39 |
| Preening | Feral (grey) | 2 | 12 | 31 |
| Preening | Feral (grey) | 2 | 18 | 11 |
| Preening | Feral (grey) | 2 | 24 | 18 |
| Preening | Feral (grey) | 2 | 30 | 48 |
| Preening | Feral (grey) | 2 | 36 | 12 |
| Preening | Feral (grey) | 2 | 42 | 47 |
| Preening | Feral (grey) | 2 | 48 | 18 |
| Preening | Feral (grey) | 3 | 6 | 50 |
| Preening | Feral (grey) | 3 | 12 | 18 |
| Preening | Feral (grey) | 3 | 18 | 10 |
| Preening | Feral (grey) | 3 | 24 | 8 |
| Preening | Feral (grey) | 3 | 30 | 6 |
| Preening | Feral (grey) | 3 | 36 | 10 |
| Preening | Feral (grey) | 3 | 42 | 6 |
| Preening | Feral (grey) | 3 | 48 | 8 |
| Preening | Feral (grey) | 4 | 6 | 33 |
| Preening | Feral (grey) | 4 | 12 | 19 |
| Preening | Feral (grey) | 4 | 18 | 46 |
| Preening | Feral (grey) | 4 | 24 | 8 |
| Preening | Feral (grey) | 4 | 30 | 25 |
| Preening | Feral (grey) | 4 | 36 | 12 |
| Preening | Feral (grey) | 4 | 42 | 35 |
| Preening | Feral (grey) | 4 | 48 | 13 |
| Preening | White Homer | 1 | 6 | 39 |
| Preening | White Homer | 1 | 12 | 22 |
| Preening | White Homer | 1 | 18 | 16 |
| Preening | White Homer | 1 | 24 | 10 |
| Preening | White Homer | 1 | 30 | 23 |
| Preening | White Homer | 1 | 36 | 15 |
| Preening | White Homer | 1 | 42 | 8 |
| Preening | White Homer | 1 | 48 | 4 |
| Preening | White Homer | 2 | 6 | 47 |
| Preening | White Homer | 2 | 12 | 49 |
| Preening | White Homer | 2 | 18 | 49 |
| Preening | White Homer | 2 | 24 | 26 |
| Preening | White Homer | 2 | 30 | 53 |
| Preening | White Homer | 2 | 36 | 50 |
| Preening | White Homer | 2 | 42 | 36 |
| Preening | White Homer | 2 | 48 | 36 |
| Preening | White Homer | 3 | 6 | 19 |
| Preening | White Homer | 3 | 12 | 50 |
| Preening | White Homer | 3 | 18 | 20 |
| Preening | White Homer | 3 | 24 | 21 |
| Preening | White Homer | 3 | 30 | 51 |
| Preening | White Homer | 3 | 36 | 50 |
| Preening | White Homer | 3 | 42 | 21 |
| Preening | White Homer | 3 | 48 | 5 |
| Preening | White Homer | 4 | 6 | 28 |
| Preening | White Homer | 4 | 12 | 16 |
| Preening | White Homer | 4 | 18 | 19 |
| Preening | White Homer | 4 | 24 | 13 |
| Preening | White Homer | 4 | 30 | 19 |
| Preening | White Homer | 4 | 36 | 8 |
| Preening | White Homer | 4 | 42 | 6 |
| Preening | White Homer | 4 | 48 | 7 |

# SUPPLEMENTARY FIGURES


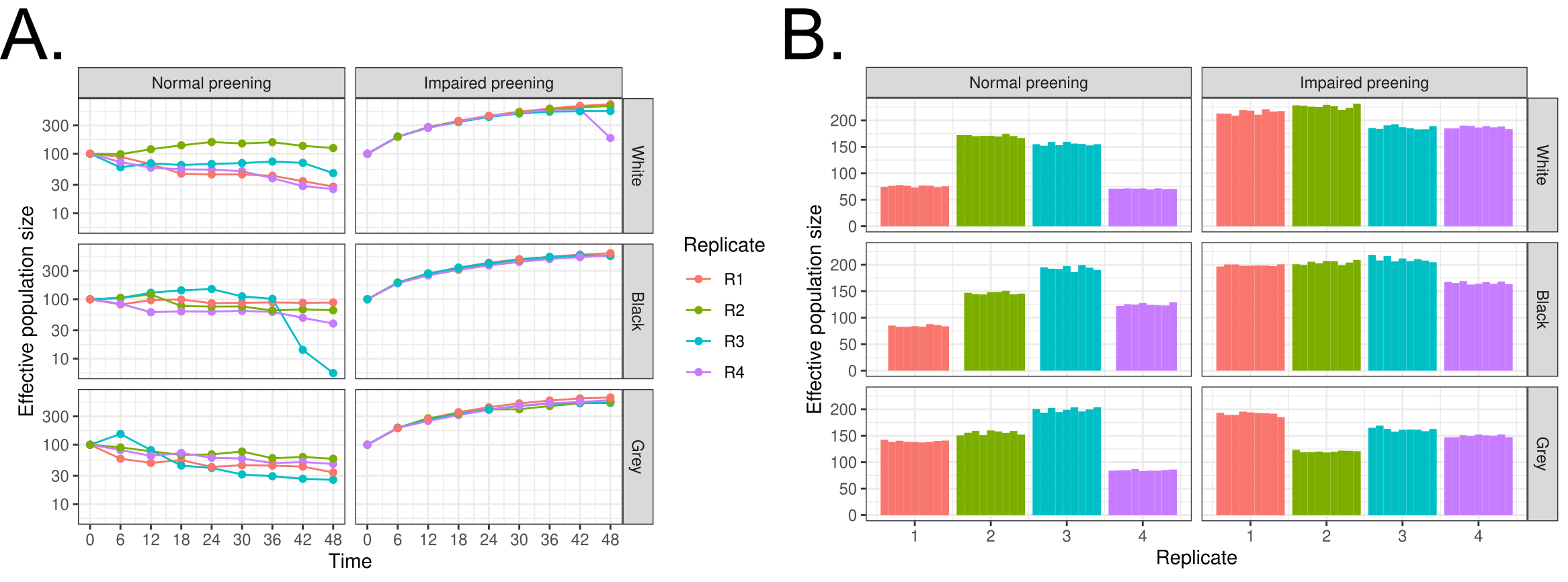


**Supplementary Figure 1.** Heterogeneity in effective population sizes across treatments is an important parameter for detecting selection. We estimated effective population size in two ways: (A) The number of lice recovered by CO_2_ fumigation in each population over the course of the experiment, corrected by harmonic mean to reflect the effective population size. (B) Effective population size, as inferred from the increase in variance of allele frequencies over time.


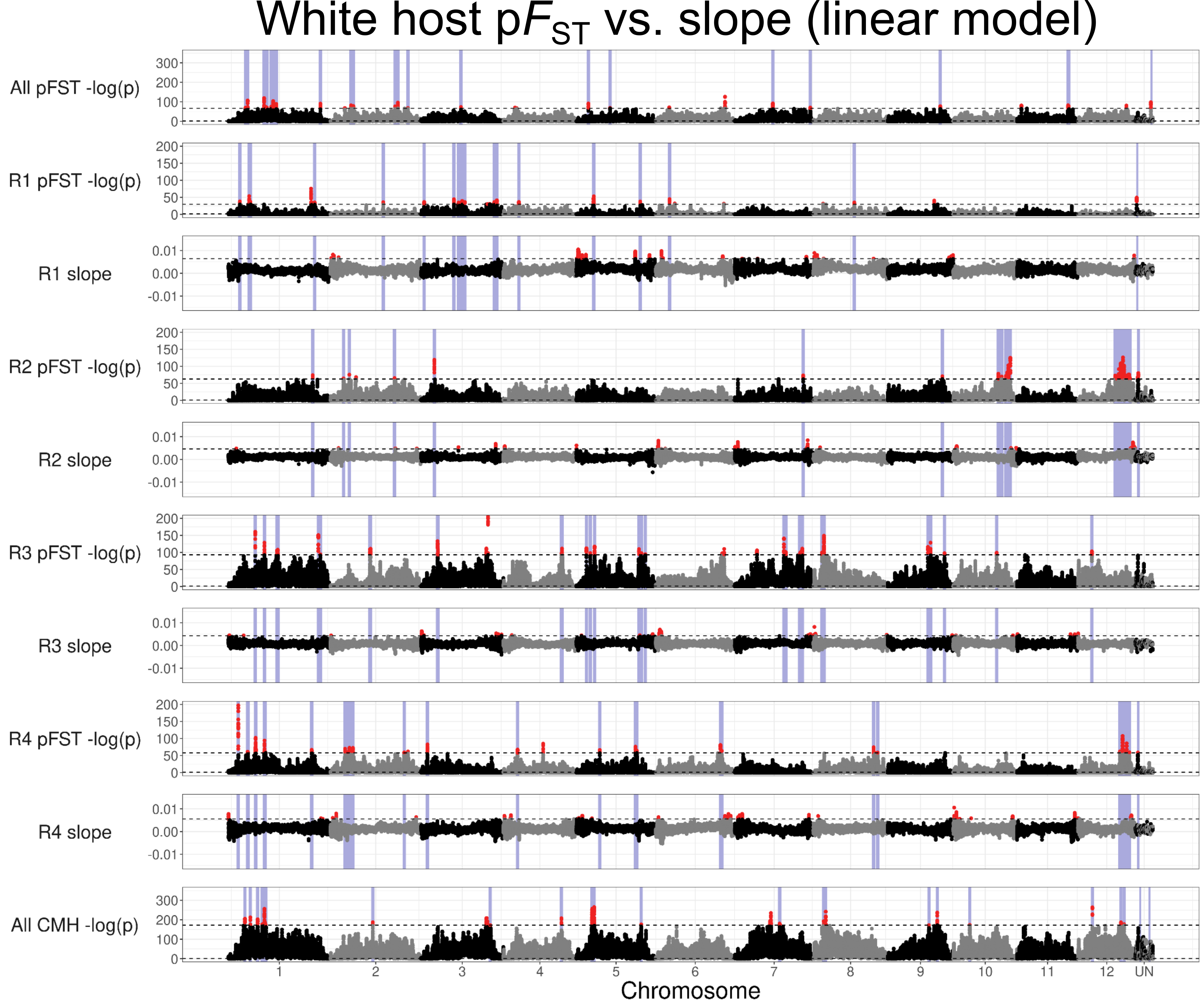


**Supplementary Figure 2.** p*F*_ST_ and estimated allele frequency slopes for evolved populations on white pigeons. Outlier allele frequency slopes largely do not match p*F*_ST_ outliers, showing that allele frequency slopes may be an effective tool for identifying selected regions missed by traditional tests of differentiation. This plot covers populations reared on white pigeons. This plot matches figure 4, but includes the 10kb-windowed allele frequency slope as well. With the caveat that slope calculations do not correct for low-coverage region, many of the outlier regions in the “slope” plots above differ from their matching “p*F*_ST_” plots.


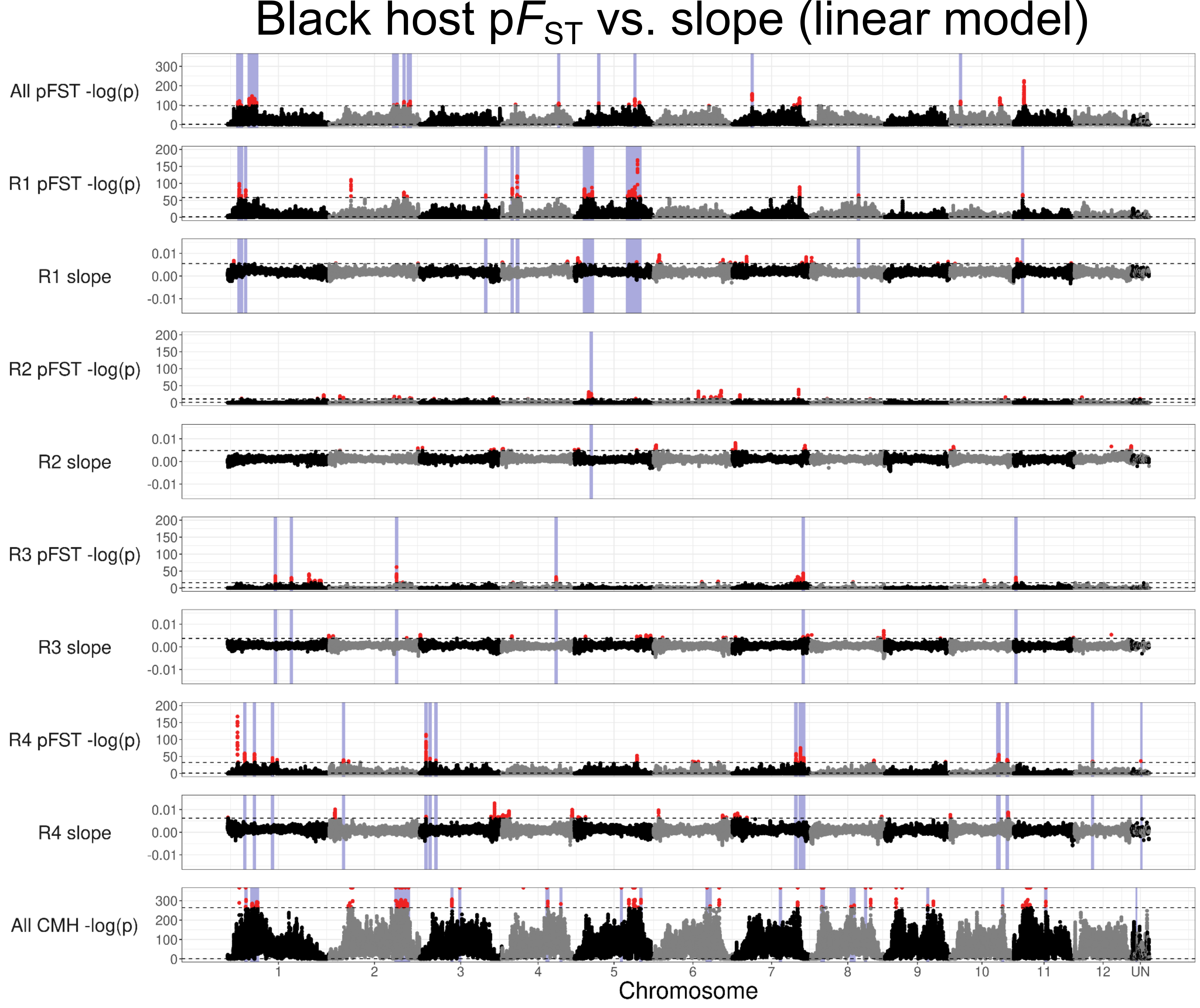


**Supplementary Figure 3.** p*F*_ST_ and estimated allele frequency slopes for evolved populations on black pigeons. Outlier allele frequency slopes largely do not match p*F*_ST_ outliers, showing that allele frequency slopes may be an effective tool for identifying selected regions missed by traditional tests of differentiation. This plot covers populations reared on black pigeons. This plot matches Figure 4, but includes the 10-kb-windowed allele frequency slope as well. With the caveat that slope calculations do not correct for low-coverage regions, many of the outlier regions in the “slope” plots above differ from their matching “p*F*_ST_” plots.


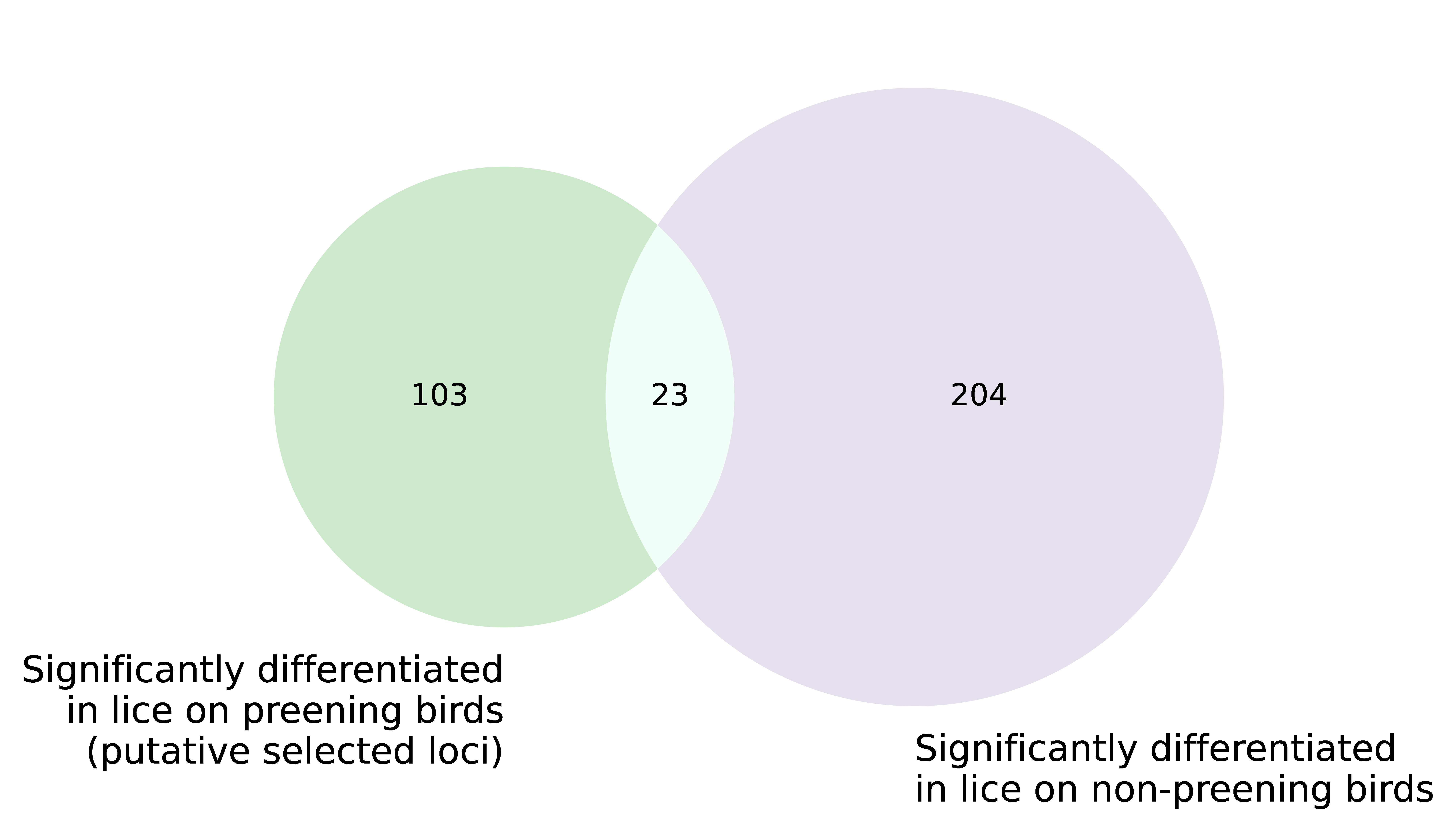


**Supplementary Figure 4.** Venn diagram depicting the overlap between loci found to be significantly differentiated in comparisonds of lice from preening vs. non-preening birds. The green circle indicates loci that were significantly differentiated in lice from at least one population on preening black or white birds when contrasted with louse populations on grey pigeons controls. The purple circle indicates loci that were significantly differentiated in the same comparison, but for louse populations on preening-impaired birds. Any loci that were identified as significant by both (the overlapping region) were excluded from further analysis because they were likely not under selection due to preening. Only the loci in the non-overlapped green circle were carried forward.


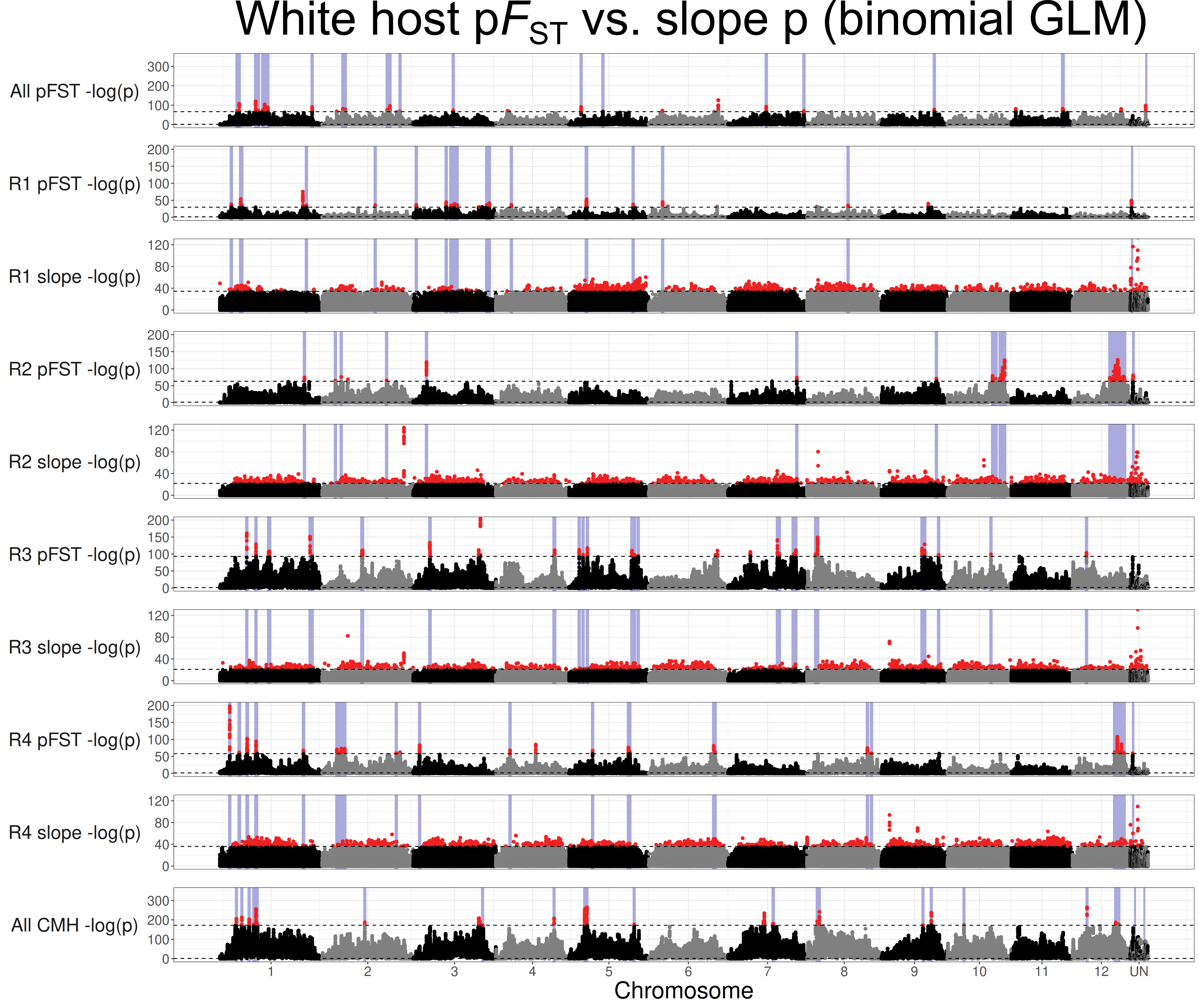


**Supplementary Figure 5.** p*F*_ST_ and binomial GLM-derived slope outlier p-values for evolved populations on white pigeons. Like the allele frequency slopes estimated by simple linear regression, outlier allele frequency slopes estimated by a binomial GLM largely do not match p*F*_ST_ outliers, showing that allele frequency slopes may be an effective tool for identifying selected regions missed by traditional tests of differentiation. This plot covers populations reared on white pigeons. This plot matches Figure 4, but includes the 10-kb-windowed allele frequency slope as well. With the caveat that slope calculations do not correct for low-coverage regions, many of the outlier regions in the “slope” plots above differ from their matching “p*F*_ST_” plots. Because the binomial GLM accounts for coverage when estimating slopes, fewer false positive outlier slopes are detected near chromosome ends.


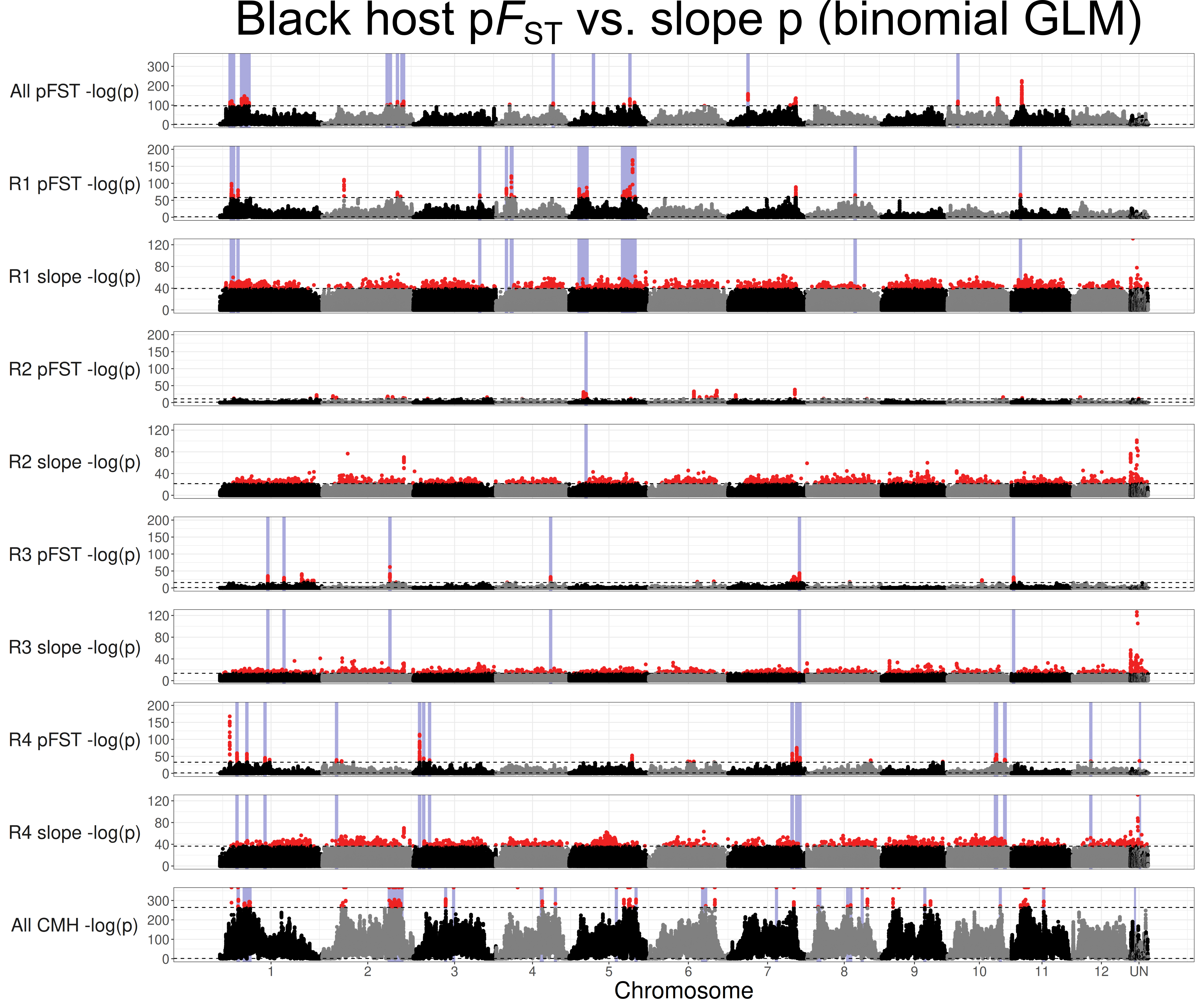


**Supplementary Figure 6.** p*F*_ST_ and binomial GLM-derived slope outlier p-values for evolved populations on black hosts. Like the allele frequency slopes estimated by simple linear regression, outlier allele frequency slopes estimated by a binomial GLM largely do not match p*F*_ST_ outliers, showing that allele frequency slopes may be an effective tool for identifying selected regions missed by traditional tests of differentiation. This plot covers populations reared on black pigeons. This plot matches Figure 4, but includes the 10-kb-windowed allele frequency slope as well. With the caveat that slope calculations do not correct for low-coverage regions, many of the outlier regions in the “slope” plots above differ from their matching “p*F*_ST_” plots. Because the binomial GLM accounts for coverage when estimating slopes, fewer false positive outlier slopes are detected near chromosome ends.

**Supplementary references**

Battey CJ, Coffing GC, Kern AD. 2021. Visualizing population structure with variational autoencoders. G3: Genes, Genomes, Genetics. 11(1):jkaa036.

Gravel S. 2016. When Is Selection Effective? Genetics 203: 451–462.

Kingma DP. 2014. Adam: A method for stochastic optimization. arXiv:14126980.
